# How virtual and mechanical coupling impact bimanual tracking

**DOI:** 10.1152/jn.00057.2022

**Published:** 2022-12-07

**Authors:** Nuria Peña-Pérez, Jonathan Eden, Ekaterina Ivanova, Ildar Farkhatdinov, Etienne Burdet

**Affiliations:** ^1^School of Electronic Engineering and Computer Science, Queen Mary University of London, London, United Kingdom; ^2^Mechanical Engineering Department, The University of Melbourne, Melbourne, Victoria, Australia; ^3^School of Engineering and Materials Science, Queen Mary University of London, London, United Kingdom; ^4^Department of Bioengineering, Imperial College of Science Technology and Medicine, London, United Kingdom

**Keywords:** bimanual, coupling, redundancy, visuomotor tracking

## Abstract

Bilateral training systems look to promote the paretic hand’s use in individuals with hemiplegia. Although this is normally achieved using mechanical coupling (i.e., a physical connection between the hands), a virtual reality system relying on virtual coupling (i.e., through a shared virtual object) would be simpler to use and prevent slacking. However, it is not clear whether different coupling modes differently impact task performance and effort distribution between the hands. We explored how 18 healthy right-handed participants changed their motor behaviors in response to the uninstructed addition of mechanical coupling, and virtual coupling using a shared cursor mapped to the average hands’ position. In a second experiment, we then studied the impact of connection stiffness on performance, perception, and effort imbalance. The results indicated that both coupling types can induce the hands to actively contribute to the task. However, the task asymmetry introduced by using a cursor mapped to either the left or right hand only modulated the hands’ contribution when not mechanically coupled. The tracking performance was similar for all coupling types, independent of the connection stiffness, although the mechanical coupling was preferred and induced the hands to move with greater correlation. These findings suggest that virtual coupling can induce the hands to actively contribute to a task in healthy participants without hindering their performance. Further investigation on the coupling types’ impact on the performance and hands’ effort distribution in patients with hemiplegia could allow for the design of simpler training systems that promote the affected hand’s use.

**NEW & NOTEWORTHY** We showed that the uninstructed addition of a virtual and/or a mechanical coupling can induce both hands to actively contribute in a continuous redundant bimanual tracking task without impacting performance. In addition, we showed that the task asymmetry can only alter the effort distribution when the hands are not connected, independent of the connection stiffness. Our findings suggest that virtual coupling could be used in the development of simpler VR-based training devices.

## Graphical abstract

**Figure:**
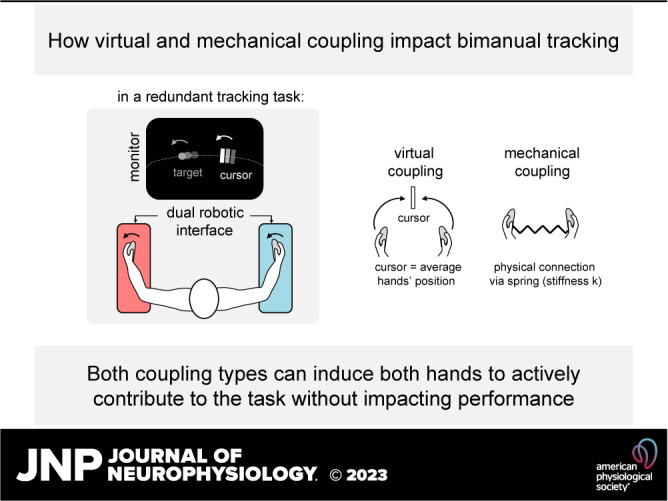

## INTRODUCTION

Many bimanual tasks, such as holding a tray or using a steering wheel, are redundant, where the same outcome can be achieved with either hand or with two hands using different coordination and effort-sharing strategies. During these tasks, cooperative action can benefit task performance. For example, the two hands can compensate for each other’s errors (1) or, as exploited by rehabilitation interfaces for hemiplegia (2, 3), one hand can take a higher share of effort. Such redundancy can be introduced into bimanual tasks by defining a common goal for the hands (4), for example by allowing them to act on the same object, which results in the hands being coupled. The coupling can be mechanical (e.g., when manipulating a physical object with the two hands) and/or virtual (e.g., when manipulating a virtual object mapped to the hands’ average position on a monitor) (5).

Activities of daily living (ADLs) typically involve mechanical coupling between the hands. Although some ADLs requiring mechanical coupling are not redundant since they limit possible coordination strategies (e.g., holding a heavy box against gravity requires a minimum force in each hand) or pre-assign hand roles (e.g., slicing bread requires one hand to cut and one to hold), other tasks, such as using a steering wheel, are fully redundant and can be performed with any effort sharing strategy between the hands. This has been used in bilateral training devices, which can provide bimanual assistance by allowing the nonaffected hand to drive the affected (2, 3, 6), where haptic feedback can facilitate performance (7). However, motor learning may be hindered by the enforcement of symmetric motions (8), or by overcompensation with the nonaffected hand (9, 10).

Virtual coupling, relying on visual feedback, can be implemented on simple virtual reality (VR) setups, and has thus been proposed for home-based rehabilitation systems (11, 12). Therefore, when developing training devices for patients with hemiplegia, an important question is whether mechanical coupling is necessary or if a virtual coupling alone is sufficient. Although a mechanical connection can provide bimanual assistance, a VR system using virtual coupling would be simpler to use and could prevent overcompensation with the nonaffected hand. In addition, it is important to understand whether the coordinated behaviors that arise during these interactions derive from the mechanical connection between the hands or are a mere result of the visualized common goal. To address these questions, it is necessary to understand the fundamental differences between these coupling modes and their impact on bimanual effort distribution and performance.

Both virtual and mechanical coupling provide information about the hands’ state that can be integrated through interhemispheric communication (13). Visual feedback of the shared object is typically available during both mechanically and virtually coupled tasks. In the case of a mechanical connection, each hand additionally receives haptic feedback from the contralateral hand. The addition of haptic feedback through a mechanical connection between the hands has been shown to improve performance during nonredundant bimanual tasks such as virtual object holding (14) and to vary with the interaction compliance (15–17). Although this has not been studied for redundant bimanual tasks, studies on common tracking during human-human interaction have found that a mechanical connection increased tracking accuracy as a result of improving sensory estimation via the exchange of haptic information (18, 19), where stiffer connections further increased tracking accuracy (20). Moreover, sensory integration models have shown that the use of multiple sensory modalities can improve performance (21).

Studies on bimanual redundant tasks suggest that participants distribute effort across the hands, where they typically act to maximize task performance with minimal effort (4, 22, 23). Stochastic optimal control has been proposed to explain this redundancy resolution (24), where a forward model estimates the system state from noisy measurements and distributes the motor commands among the available end-effectors to minimize error and effort (25). This framework predicts the central nervous system’s (CNS’s) observed behavior of minimizing task-relevant variability without unnecessarily exerting effort when it is task irrelevant. For bimanual coordination, this means that when a clear source of task-relevant variability is introduced (e.g., by perturbing one hand), if the hands are coupled, either virtually (4, 22, 26) or mechanically (27), they will both engage in “optimal” corrective motions. This however relies on the assumption that participants can recognize task-relevant feedback modalities. While initial findings suggest that a lack of explicit instructions does not prevent participants from adapting differently to task-relevant and irrelevant feedback [e.g., adapting to altered weightings of a shared cursor during bimanual reaching (28)], it is unclear if such adaptation is possible during continuous bimanual tasks. For instance, task-irrelevant motions were not minimized in a planar tracking task where the hands were split to control different degrees of freedom (29).

Lateralization has been found to influence hand effort distribution during bimanual redundant tasks. In virtually coupled isometric tasks, the nondominant hand has been observed to contribute less to the task than the dominant hand (30), supporting previous studies that showed that the different contributions stem from the respective noise properties (25). These contribution asymmetries are however affected by factors such as movement direction and age (30), posture (31), temporal demands (29), and the provided sensory feedback (32–34). Instead, in the mechanical coupling, lateralization has been mostly studied in (right-handers for) nonredundant tasks, where rather than effort distribution, differences in hand control properties were studied. Here, it has been suggested that each hand specializes in different control aspects, where the dominant hand would perform finer controlled motions whereas the nondominant hand would provide stability against environmental disturbances. This has been reported in asymmetric tasks (35) but has been shown to depend on factors such as age (36) and symmetry requirements (37, 38).

We conducted a study to explore if the type of coupling impacts how humans distribute the effort among their hands in a continuous redundant task, and how it affects their performance and perception. Healthy right-handed participants controlled a single cursor in a one-degree-of-freedom tracking task by performing flexion/extension motions of the two wrists. We first explored how 18 participants changed their motor behaviors in response to the uninstructed addition of a medium-hard (39) virtual spring connecting the hands, a virtual coupling through shared visual feedback (with equal cursor weighting reflecting the hands’ average position vs. unequal weighting using either the left or right hand position), and the combination of both. In a second experiment, we then investigated whether the effort imbalance changes with the asymmetry introduced by unequal weighting for different connection stiffness and how these impact performance. Here, four groups of ten participants each performed the same tracking task with a different connection stiffness.

We hypothesized that “participants would not use a hand if it does not impact the task (*H1*),” using both hands only when they are coupled (either virtually or mechanically). However, we expected participants to “use different effort contributions across the different conditions (*H2*).” In particular, we hypothesized that the contribution of the hands would not be balanced when they are virtually coupled, where the addition of a mechanical connection would introduce reaction forces that could result in balanced effort distributions. Moreover, we expected unequal cursor weightings to also lead to unbalanced effort contributions, caused by either the higher reliability of one hand or its different functional role. Furthermore, we hypothesized that “the additional haptic feedback received during mechanical coupling would benefit tracking performance, where the tracking accuracy would improve with increasing connection stiffness (*H3*).”

## MATERIALS AND METHODS

### Participants

**Figure 1.**
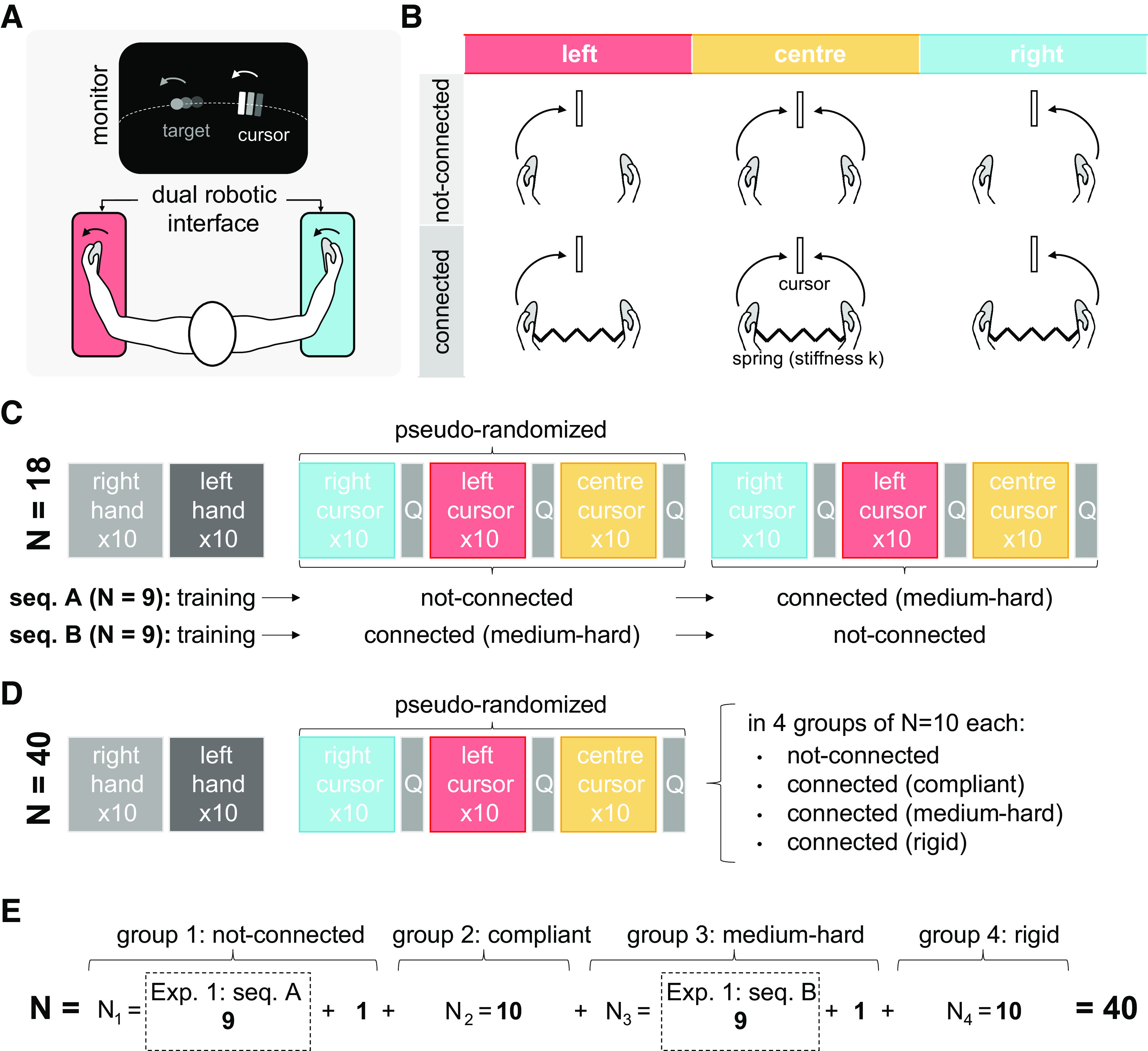
Experiment setup, task conditions, and protocols. *A*: participants sat in front of a monitor visualizing a single (5 × 95 pixel rectangle) cursor and the (14 pixel diameter circle) target and held one handle of a dual robotic interface with each hand. Since the cursor and target motion was constrained to a 1,700-pixel arc, the target diameter was equivalent to 1° of motion, and the cursor’s width covered one third of it. The visualized trajectory of the target was constrained to lie on that arc in the angular range [−28.2, 25.7]°. *B*: the cursor’s motion was mapped to either their left wrist position, the right or their average (center) depending on the experimental block while their hands could be either not-connected or connected through a mechanical connection of stiffness *K*. Protocols for *experiment 1* (*C*), where all 18 participants tried the three cursor weightings with the hands not-connected and connected through a medium-hard virtual spring (*K* = 2.86 Nm/rad) in either of two sequences and *experiment 2* (*D*), where the 40 participants were split in four groups of ten, each performing the three cursor weightings with a connection level: not-connected, compliant (0.63 Nm/rad), medium-hard (2.86 Nm/rad) or connected through a rigid bar. The cursor weighting order was always pseudo-randomized. Participants started with the training phase and between experimental blocks, they answered a series of questions (Q). *E*: *experiment 1* data (from experimental blocks 1–3) was split into two groups of nine based on the participant’s sequence. In addition, data from 22 new participants was collected, two participants to complete the groups of nine and 20 for the two new groups.

The experiments were granted ethical approval by the Joint Research Compliance Office at Imperial College London (Reference 15IC2470). *Experiment 1* was carried out by 18 healthy participants (nine female and nine male), aged 21–34 yr (mean = 26.11, SD = 3.32). *Experiment 2* analyzed data from a total of 40 participants (15 female and 25 male), aged 20–46 (mean = 25.02, SD = 4.72), who were allocated across four groups of ten participants each. For this experiment, data from *experiment 1* was split into two equal groups of nine based on the participant’s sequence (Fig. 1*C*). In addition, data from 22 new participants were collected, including 2 participants to complete the groups of 9 and 20 for the 2 additional groups (Fig. 1*E*). All participants were naïve about the experimental conditions and gave their written informed consent before starting the experiment. The handedness of each participant was determined using the Edinburgh Handedness Inventory (40) and their laterality quotient (LQ) was calculated (where LQ = −100 is extreme left handedness and LQ = 100 extreme right handedness). All participants were right handed with LQ *>* 70 (*experiment 1*: mean = 98.5, SD = 6.36; *experiment 2*: mean = 97.72, SD = 6.34).

### Experimental Setup

A tracking experiment was conducted using the *Hi5* dual robotic interface (41) illustrated in Fig. 1*A*. This one-degree-of-freedom robotic interface enables the study of coordinated flexion/extension movements of two wrists by measuring the angle, torque, and activity of flexor and extensor muscles. Hi5’s handles can be mechanically coupled through a physical rigid bar or via a virtual spring generated using computer-controlled torque on each wrist. The interface was controlled at 1,000 Hz, while wrist angle data were recorded at 100 Hz. Surface electromyography (EMG) from the wrist flexor carpi radialis (FCR) and extensor carpi radialis longus (ECRL) muscles in the left and the right wrists was recorded at 1,000 Hz using the g.GAMMASYS system (g.tec).

### Tracking Task

Participants were asked to control a single cursor on a monitor using their wrist flexion/extension and to track a moving target “as accurately as possible.” In this way, their visual display was always that of Fig. 1*A*, such that their right-wrist flexion or their left-wrist extension would move the controlled cursor in the anticlockwise direction. Depending on the experimental condition (Fig. 1*B*), the cursor’s position (*q*) was controlled with a direct mapping of the left-wrist position (left weighting: *q* = *q*_l_), the right-wrist position (right weighting: *q* = *q*_r_), or with their average position [center weighting: *q* = (*q*_r_ + *q*_l_)/2]. In this way, the center condition used equal hand weighting, whereas the right and left conditions used unequal weighting.

The target trajectory (in degrees) was given by the following multisine function:

$$q^{*}\left(t\right)=-7.8\text{sin}\left(0.48t^{*}\right)+1.6\text{sin}\left(1.12t^{*}\right)+9.4\text{sin}\left(1.48t^{*}\right)-10.6\text{sin}\left(2.56t^{*}\right),t^{*}=t+t_{0},0\;\leq t\leq \text{ 25 s}.$$

Each trial started from a randomly selected starting time {*t*_0_ ∈ [0,25]s|*q*^∗^(*t*_0_) ≡ 0} to minimize learning of the trajectory.

During *experiment 1*, the hands were either not connected or mechanically connected through a virtual spring of medium-hard stiffness (2.86 Nm/rad), chosen based on previous human interaction work which found that this stiffness can be clearly perceived by participants while still allowing for some flexibility (39). During *experiment 2*, the hands could also be connected by a compliant virtual spring (0.63 Nm/rad) (39) or a physical rigid bar.

### Experimental Protocol

The experimental protocols are depicted in Fig.1 *C* and *D*. Each participant started with a training phase in which they had to track the moving target first with their right hand and then with their left hand, for ten trials each, while the cursor was set to show the relevant hand’s position.

Two factors were explored in both experiments. The first factor was the cursor weighting, with three within-subject levels: the equal weighting condition, which introduced the virtual coupling, and the right and left unequal weighting conditions introducing task asymmetry. The second factor was the connection that had two within-subject levels for *experiment 1* and four between-subject levels for *experiment 2*. This resulted in six experimental conditions for *experiment 1* (Fig. 1*C*) and three for each participant in *experiment 2* (Fig. 1*D*). During the testing phase, the corresponding experimental conditions were presented in blocks of ten trials each. Participants were told that they could choose to use their hands individually or concurrently, but they were not given any other instructions. After each block, a short series of questions was presented to the participants (see questionnaire in Supplemental Section 3.1, all Supplemental materials are available at https://doi.org/10.6084/m9.figshare.21370950). During *experiment 1*, the sequence of the connected/nonconnected blocks was counterbalanced among participants, with a pseudorandom order of the cursor conditions in both experiments.

### Data Analysis

EMG activity was high-pass filtered with a 20-Hz cutoff frequency, rectified and then low-pass filtered with a 5-Hz cutoff frequency (all second-order Butterworth filters). The activity of the wrists’ flexor and extensor muscles, measured in volts, was calibrated by linearly regressing the activity of each muscle with the torque (in Nm) produced by the muscle during isometric contraction (41).

To assess whether participants used their hands in a task-relevant way (*Hypothesis H1*), we examined how much they moved each wrist compared with the target’s motion. The normalized arc-length (NAL) was computed for each trial as the arc-length of the wrist’s trajectory (*q*_l_ or *q*_r_) divided by that of the target’s trajectory (q*), such that values higher than 1 would imply that in that trial the wrist moved more than the target, whereas values lower than 1 would mean that the wrist moved less than required.

To evaluate whether the hands contributed differently across conditions and whether both hands contributed equally in each condition (*Hypothesis H2*), two metrics were calculated from the torque-normalized EMG. First, effort contributing to motion was calculated for each wrist as the absolute reciprocal flexor and extensor activation (RA), where *u*_ra_(*t*) ≡ max{|τ_f_(*t*)|,|τ_e_(*t*)|} − min{|*τ*_f_(*t*)|,|*τ*_e_(*t*)|}. Second, the co-contraction (CC) of each wrist was computed as the minimum overlapping flexor and extensor torque (*u*_cc_(*t*) ≡ min{|τ_f_(*t*)|,|τ_e_(*t*)|}). Furthermore, to explore whether the difference in the two hands’ contributions was impacted by the asymmetry introduced by unequal cursor weightings, we calculated the NAL, RA, and CC imbalance (as the difference between the left and right hand’s value for each trial).

Last, to evaluate whether the connection stiffness influenced the performance (*Hypothesis H3*), the tracking error was computed as the root mean squared (RMS) error between the controlled cursor’s motion and the target. In addition, we evaluated how participants perceived the physical connection (question Q5, see Supplemental Section 3.1) and whether the hands were consistently moving together in each trial through the Spearman correlation between the wrists’ positions (due to the non-normality of the wrist position data).

After preprocessing in MATLAB, data were analyzed using RStudio. To focus on the tracking behavior, data in the first second of every trial were removed to account for different reaction times. To determine if participants adjusted their performance within each block, the tracking error tendency along the first five and the last five trials of each condition was explored using linear mixed effects (LME) analysis via restricted maximum likelihood (RML), with the trial number as a fixed slope (*s*) and a random intercept for each grouping factor (subject ID). The Satterthwaite method was used to calculate an approximation for the degrees of freedom. The performance during the last five trials of each experimental condition was found to no longer be significantly decreasing, as indicated by nonsignificant slopes (all *P* > 0.08). For this reason and to focus on steady-state behaviors, for the statistical analysis we used only the data averaged for each participant across the last five trials of each block (further information in Supplemental Section 2.2).

### Statistical Analysis

Normality was checked by performing Shapiro–Wilk tests. Given that not-normally distributed conditions were found in all metrics, nonparametric analysis was used on the data.

The influence of the two factors (i.e., cursor weighting and connection) on the tracking error during the test phase, the correlation between the hands, and the subjective assessment on the perception of the physical connection were explored using two-way Aligned Rank Transformed (ART) ANOVA (42), repeated measures for *experiment 1* and mixed for *experiment 2*. Here, *Hypothesis H3* could be confirmed by either a main effect of the connection or a significant interaction, with better performance for stronger connection stiffness for at least the center cursor condition. In addition, to assess the initial unimanual skill level, the performance during the left and right training blocks was explored through a Wilcoxon paired test.

In *experiment 1*, the NAL, RA, and CC were explored through repeated measures three-way ART ANOVA with the “hand” as an additional factor. *Hypothesis H1* could be confirmed by a significant interaction of the three factors in the NAL, where differences between the hands would only be found in the uncoupled cases, and where the “non-relevant” hand would move less in the uncoupled conditions compared with the coupled. A three-way interaction in the RA and CC analysis, with differences between hands during virtual coupling and not during mechanical coupling, could confirm *Hypothesis H2*.

Moreover, to explore whether the effort imbalance depends on the asymmetry introduced by unequal cursor weightings for different values of connection stiffness, the NAL, RA, and CC imbalances were explored through LME analysis via RML in *experiment 2*. Here, we used a random intercept for each grouping factor (subject ID) and the cursor weighting as a fixed slope (*s*), such that the center condition was considered to be zero, and the right was considered positive (with a value of one). Here, as per *Hypothesis H2*, a significant slope would suggest that the effort imbalance depends on the cursor weighting.

Post hoc analysis was conducted by performing a series of tailored pairwise comparisons: *1*) within-subject differences among cursor weighting levels for each connection level; *2*) within- or between-subject differences across connection levels for each cursor weighting level, and *3*) left versus right-hand comparisons for each of the six combinations of cursor weighting and connection levels (whenever the “hand” factor was used). Wilcoxon paired tests were used for comparisons within subjects and Mann–Whitney tests for comparisons between subjects.

*P* values were adjusted using the Hommel or the Benjamini–Hochberg correction (when the number of comparisons was higher than 24) to control for type I error in multiple comparisons. The level of significance was set at α = 0.05 and any *P* values smaller than 0.001 are reported as *P* < 0.001.

The presented figures show all the observed significant differences, while the most relevant results are reported in the text. It should be noted that main effects are only reported whenever a significant interaction was not observed.

## RESULTS

### Experiment 1: Does the Coupling Type Impact the Effort Distribution, Performance, and Perception?

#### When virtually or mechanically coupled, the hands contributed similarly to the task.

##### Most participants used their hands in a task-relevant way (H1).

**Figure 2.**
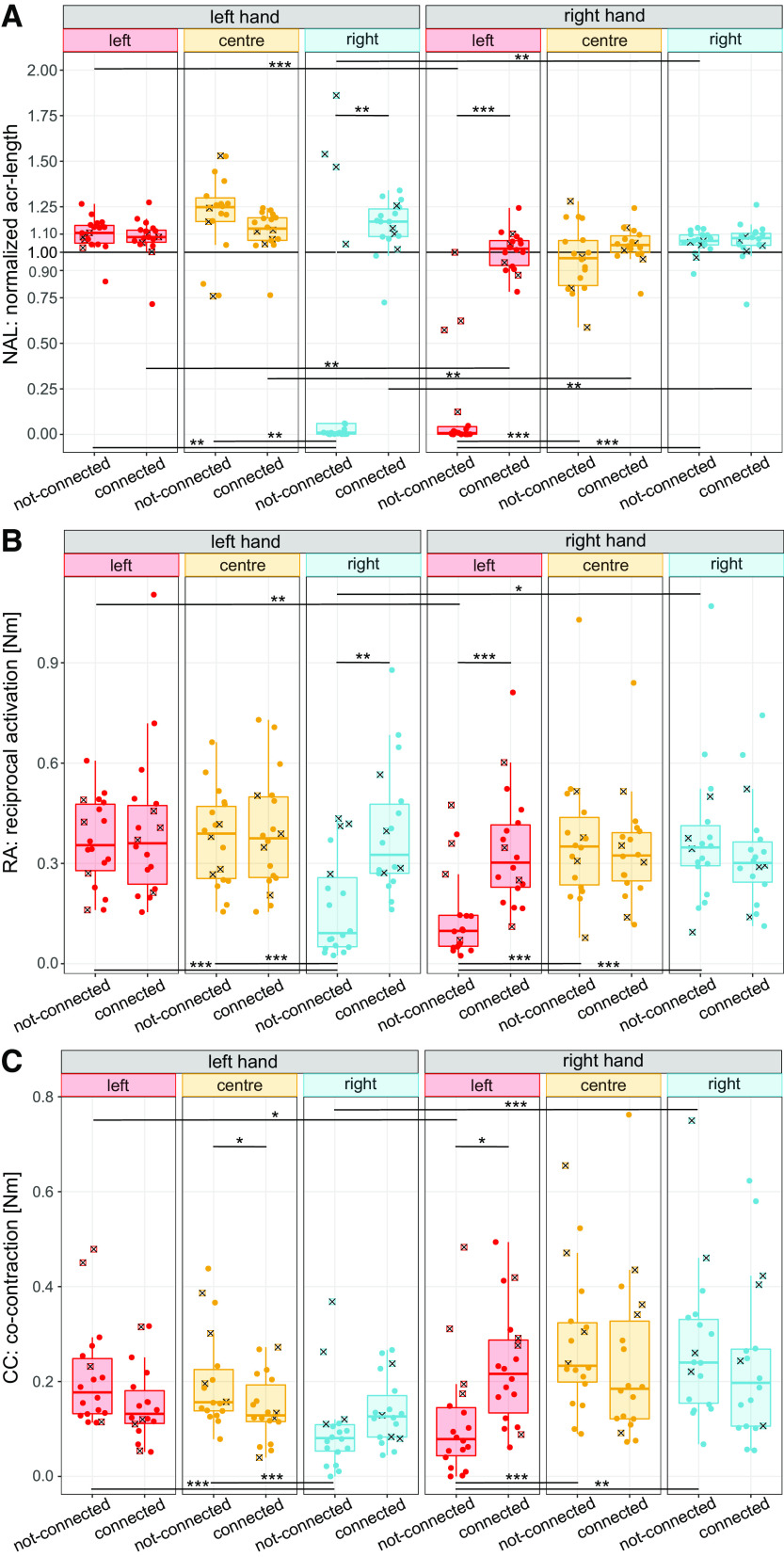
*Experiment 1*: normalized arc-length (*A*), effort spent in motion (*B*), and co-contraction (*C*) for each experimental condition where each dot is the mean across the last five trials per participant. Squared-crossed markers represent participants from the “atypical” subset. *P < 0.05, **P < 0.01, ***P < 0.001. Comparisons not shown are not significant.

The normalized arc-length (NAL) showed a significant interaction of the cursor weighting, connection, and hand [*F*(2,34) = 81.37, *P* < 0.001]. Despite the lack of explicit instructions, most participants moved both hands for all coupled conditions, but only the task-relevant hand in the uncoupled conditions (Fig. 2*A*).

In this way, the right hand moved less than the left hand during the not-connected-left condition (*W* = 171, *Z* = −3.76, *P* < 0.001) and showed less motion than in the not-connected-center (*W* = 3, *Z* = −3.35, *P* < 0.001), not-connected-right (*W* = 0, *Z* = −3.76, *P* < 0.001) and connected-left (*W* = 0, *Z* = −3.76, *P* < 0.001) conditions. Similarly, the left hand moved less than the right during the not-connected-right condition (*W* = 10, *Z* = −2.81, *P* = 0.005) and showed less motion than in the not-connected-center (*W* = 161, *Z* = −2.81, *P* = 0.005), not-connected-left (*W* = 162, *Z* = −2.87, *P* = 0.004) and connected-right (*W* = 11, *Z* = −2.73, *P* = 0.006) conditions. This suggests that most participants identified differences in the feedback received and changed their motor behavior consequently.

**Figure 3.**
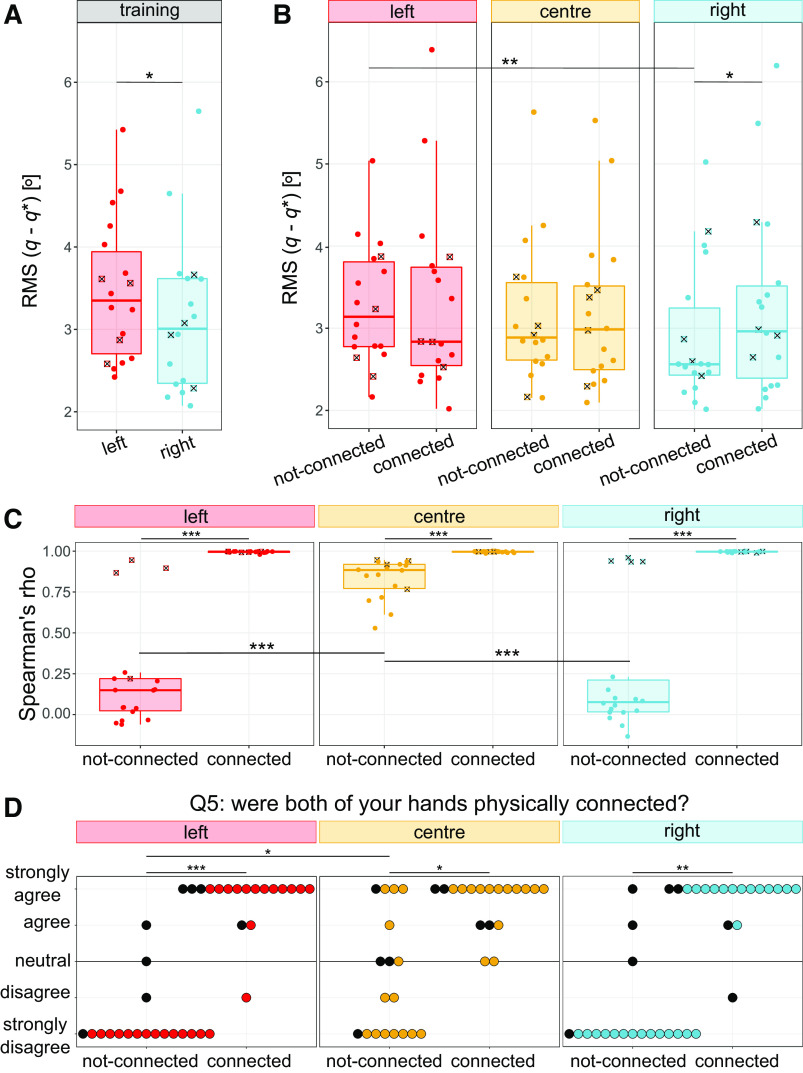
*Experiment 1*: Tracking error for the training (*A*) and test phases (*B*) and correlation (*C*) between the hands for each experimental condition (where each dot is the mean across the last five trials per participant). *D*: perception of the connection. Squared-crossed markers (*A*–*C*) and black dots (*D*) represent participants from the “atypical” subset. *P < 0.05, **P < 0.01, ***P < 0.001. Comparisons not shown are not significant. RMS, root mean square.

However, it can be observed that a subset of four participants (who will be referred as “atypical” participants) did move their left hand during the not-connected-right condition, with three of them also moving their right hand in the not-connected-left condition (Fig. 2*A*). Note that given they were not outliers in any other condition nor showed a qualitatively different performance (Fig. 3, *A* and *B*), all participant data were included in the analysis. These differences are consistent with the intratrial tendencies observed in Supplemental Fig. S3, where 14 out of the 18 participants moved both hands when they were virtually and/or mechanically coupled and used only the relevant hand when the coupling was removed. In contrast, these 4/18 participants exhibited an “atypical” behavior, simultaneously moving both hands in the not-connected-left and the not-connected-right blocks.

When the hands were mechanically connected, the amount of motion of the left hand was closer to the target during the left cursor condition compared with the center (*W* = 18, *Z* = −2.23, *P* = 0.026), with no differences being observed for either hand for the remaining conditions (all *P* > 0.05). Interestingly, the amount of motion of the left hand was consistently higher than the right hand’s for all of the mechanically connected conditions (connected-left: *W* = 157, *Z* = −2.52*, P* = 0.012; connected-center: *W* = 164*, Z* = −3.03*, P* = 0.002; connected-right: *W* = 159*, Z* = −2.67*, P* = 0.007), but no difference between the hands was found during the virtual coupling (*W* = 137*, Z* = −1.56*, P* = 0.12).

##### The effort distribution was balanced between the two hands in all coupled conditions (H2).

A significant interaction of the cursor weighting, connection, and hand was found for the RA [*F*(2,34) = 29.72*, P* < 0.001] and the CC [*F*(2,34) = 40.32*, P* < 0.001]. In this way, although the left hand tended to spend more effort (higher RA) and the right hand tended to be more co-contracted (Fig. 2, *B* and *C*), unbalanced effort distributions were only observed when the hands were uncoupled.

The balanced effort contributions were confirmed by the lack of differences between the hands once they were coupled (all *P* > 0.1). Instead, if a hand could not impact the cursor its contribution was lower than that of the other hand: the right hand’s was lower during the not-connected-left condition (RA: *W* = 166*, Z* = −3.15*, P* = 0.002, CC: *W* = 155*, Z* = −2.29*, P* = 0.022) and the left hand’s was lower during the not-connected-right condition (RA: *W* = 14*, Z* = −2.45*, P* = 0.014, CC: *W* = 1*, Z* = −3.58*, P* < 0.001).

In line with the NAL (Fig. 2*A*) and the intratrial trajectories (Supplemental Fig. S3), during the not-connected conditions, any increase in the cursor weighting contribution of a hand increased its effort, both in terms of the RA and the CC. This was confirmed by *1*) the lower effort of the right hand in the not-connected-left when compared with the virtual coupling (RA: *W* = 0*, Z* = −3.74*, P* < 0.001 and CC: *W* = 3*, Z* = −3.35*, P* < 0.001) and the not-connected-right (RA: *W* = 1*, Z* = −3.57*, P* < 0.001, CC: *W* = 5*, Z* = −3.17*, P* = 0.002) and *2*) the lower effort of the left hand in the not-connected-right when compared with the not-connected-left (RA: *W* = 158*, Z* = −2.53*, P* = 0.011, CC: *W* = 170*, Z* = −3.58*, P* < 0.001) and the virtual coupling (RA: *W* = 159*, Z* = −2.61*, P* = 0.009, CC: *W* = 171*, Z* = −3.75*, P* < 0.001). However, once the hands were mechanically coupled, introducing asymmetry by changing the cursor weighting did not have any effect on either the RA (all *P* > 0.1) or the CC (all *P* > 0.6).

Similar to the virtual coupling, the mechanical connection also induced the left hand to actively participate in the task, however, the virtual coupling may have been more efficient at increasing its CC. The mechanical connection increased the RA of the left hand with the right cursor weighting (*W* = 9*, Z* = −2.82*, P* = 0.005), but this increase in motion-related effort was not accompanied by an increase in CC (*W* = 38*, Z* = −0.94*, P* = 0.35). Moreover, during the center condition, the left hand was less co-contracted when mechanically coupled than when virtually coupled to the right hand (*W* = 152*, Z* = −2.06*, P* = 0.04). Instead, the effort of the right hand with the left cursor weighting was increased by mechanically connecting the hands both in terms of the RA (*W* = 3*, Z* = −3.34*, P* < 0.001) and CC (*W* = 19*, Z* = −2.06*, P* = 0.04).

#### The coupling types did not affect tracking performance, but were perceived differently and induced different behaviors.

##### Participants could track the target equally well in all coupled conditions (H3).

Although the interaction between the cursor weighting and connection was found to impact the tracking accuracy [*F*(2,34) = 7.75*, P* = 0.002], the addition of a mechanical connection to a virtual coupling did not improve performance (Fig. 3*B*). Moreover, once the hands were mechanically coupled the tracking accuracy was not altered by changes in the cursor weighting.

In this way, the tracking accuracy was similar in all coupled conditions (all *P* > 0.07). The tracking error was however lower in the not-connected-right condition compared with the not-connected-left (*W* = 153*, Z* = −2.61*, P* = 0.009) and the connected-right (*W* = 19*, Z* = −2.5*, P* = 0.019), with participants also tracking more accurately during the right hand’s training than during the left’s (*W* = 140*, Z* = −2.41*, P* = 0.016, see Fig. 3*A*). This indicates that while participants tracked more accurately when performing dominant unimanual motions compared with nondominant ones, their performance was unchanged once the hands were coupled.

Participants solved the task differently under different coupling types with more correlated motions during the mechanical coupling. The interaction of the cursor weighting and connection significantly impacted the correlation between the hands [*F*(2,34) = 75.69*, P* < 0.001] with the mechanical connection improving the correlation between the hands for all cursor weightings (all *P* < 0.001), including when compared with the virtual coupling. The virtual coupling did however improve the correlation between the hands compared with the not-connected-left (*W* = 3*, Z* = −3.69*, P* < 0.001) and not-connected-right (*W* = 164*, Z* = −3.38*, P* < 0.001) conditions.

Although these results indicate that both mechanical and virtual coupling can each alter correlation, the cursor weighting did not have any effect on the correlation between the hands (all *P* > 0.3, Fig. 3*C*) while they were mechanically connected. This suggests that once the mechanical connection is present, an equal cursor weighting does not further improve the correlation.

##### The mechanical connection was clearly perceived.

Responses to “both of my hands were physically connected” (Fig. 3*D*) exhibited a significant interaction of the cursor weighting and connection [*F*(2,34) = 8.63*, P* < 0.001]. Participants had a stronger perception of a physical connection between their hands when they were mechanically connected, for all cursor weightings (left: *W* = 0*, Z* = −3.33*, P* < 0.001; center: *W* = 11.5*, Z* = −2.45*, P* = 0.014; right: *W* = 2*, Z* = −3.19*, P* = 0.001). Interestingly, participants had a stronger sense of connection when the hands were virtually coupled compared with the not-connected-left condition (*W* = 0*, Z* = −1.98*, P* = 0.048).

### Experiment 2: How Does the Connection Stiffness Affect the Effort Imbalance and Performance?

#### The effort imbalance was unaltered by the cursor weighting for all connection stiffness levels.

##### Unequal cursor weightings only modulated the effort imbalance when the hands were not mechanically connected (H2).

This was revealed by a significant negative slope for the not-connected group (RA imbalance: *s* = −0.15*, t*(19) = −4.00*, P* < 0.001; CC imbalance: *s* = −0.12*, t*(19) = −4.42*, P* < 0.001) and non-significant slopes for all mechanically connected groups (all *P* > 0.37). The same result was found for the amount of motion of each hand (NAL imbalance: *s* = −0.68, *t*(28) = −6.10*, P* < 0.001).

##### The hands contributed differently when compliantly connected.

**Figure 4.**
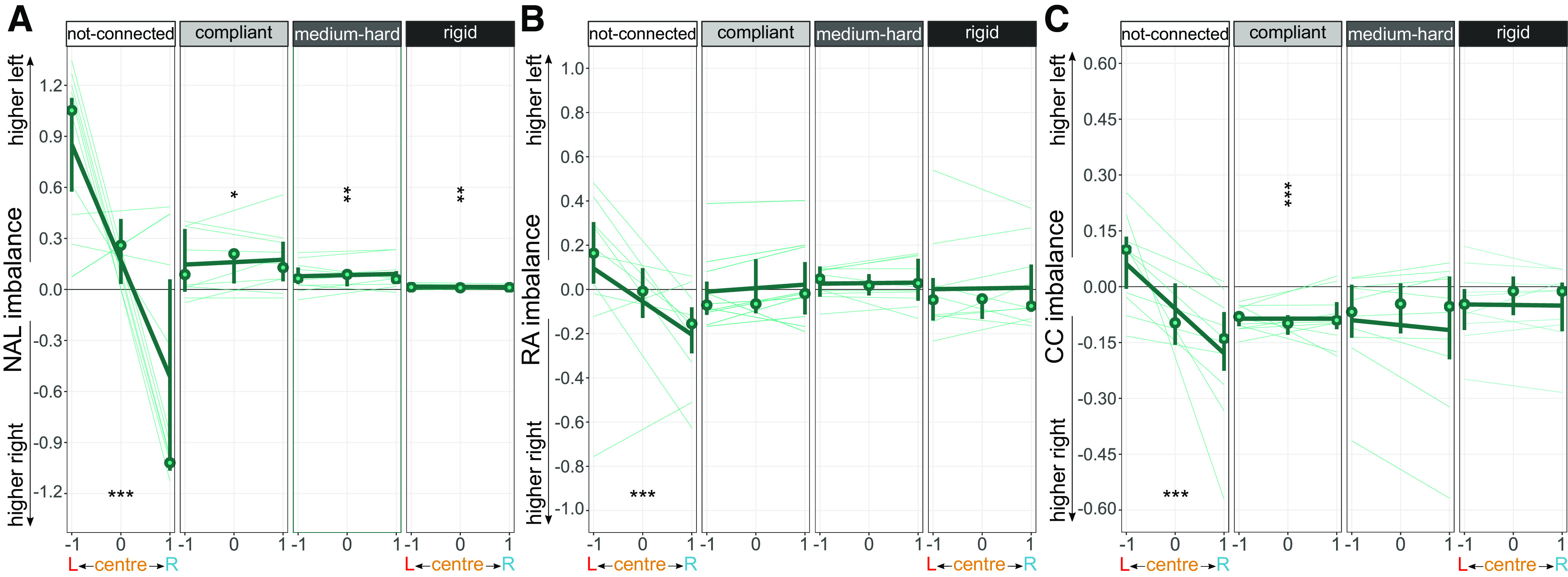
Effort imbalance in *experiment 2*, where positive values correspond to a higher contribution of the left hand, and negative values correspond to a higher right hand’s contribution. Linear mixed effect models were fit to the normalized arc-length imbalance (*A*), reciprocal activation imbalance (*B*), and the co-contraction imbalance to explore the effect of the changing cursor weighting on the imbalance (*C*). The hands had a shared (zero) influence on the cursor during the center condition. Significant slopes are displayed with horizontal markers and significant intercepts are displayed with vertical markers. *P < 0.05, **P < 0.01, ***P < 0.001.

As expected from the findings of *experiment 1*, the effort imbalance of the virtually coupled and medium-hard connection groups was close to zero, with nonsignificant intercepts (all *P* > 0.08), suggesting similar hands’ contributions (see Fig. 4, *B* and *C*). However, while similar results were found for the rigid group (*P* > 0.15 for both the RA and the CC), participants with a compliant connection were found to co-contract their right hand more than their left [negative significant intercept: *b* = −0.09*, t*(9) = −6.07*, P* < 0.001], while keeping a balanced RA [*b* = 0.006*, t*(9) = 0.11*, P* = 0.92].

As found in *experiment 1*, analysis of the NAL imbalance showed that participants who had their hands mechanically connected moved their left hand more than the right (Fig. 4*A*). This was independent of the connection stiffness [positive significant intercepts, compliant: *b* = 0.16*, t*(9) = 2.90*, P* = 0.018; medium-hard: *b* = 0.08*, t*(8.99) = 3.27*, P* = 0.010; rigid: *b* = 0.01*, t*(8.99) = 3.93*, P* = 0.004] and not observed during the virtual coupling [nonsignificant intercept: *b* = 0.17*, t*(28) = 1.87*, P* = 0.07.

#### Connection stiffness did not alter the tracking error but affected the behavior and perception.

##### The tracking did not improve with larger stiffness (H3).

**Figure 5.**
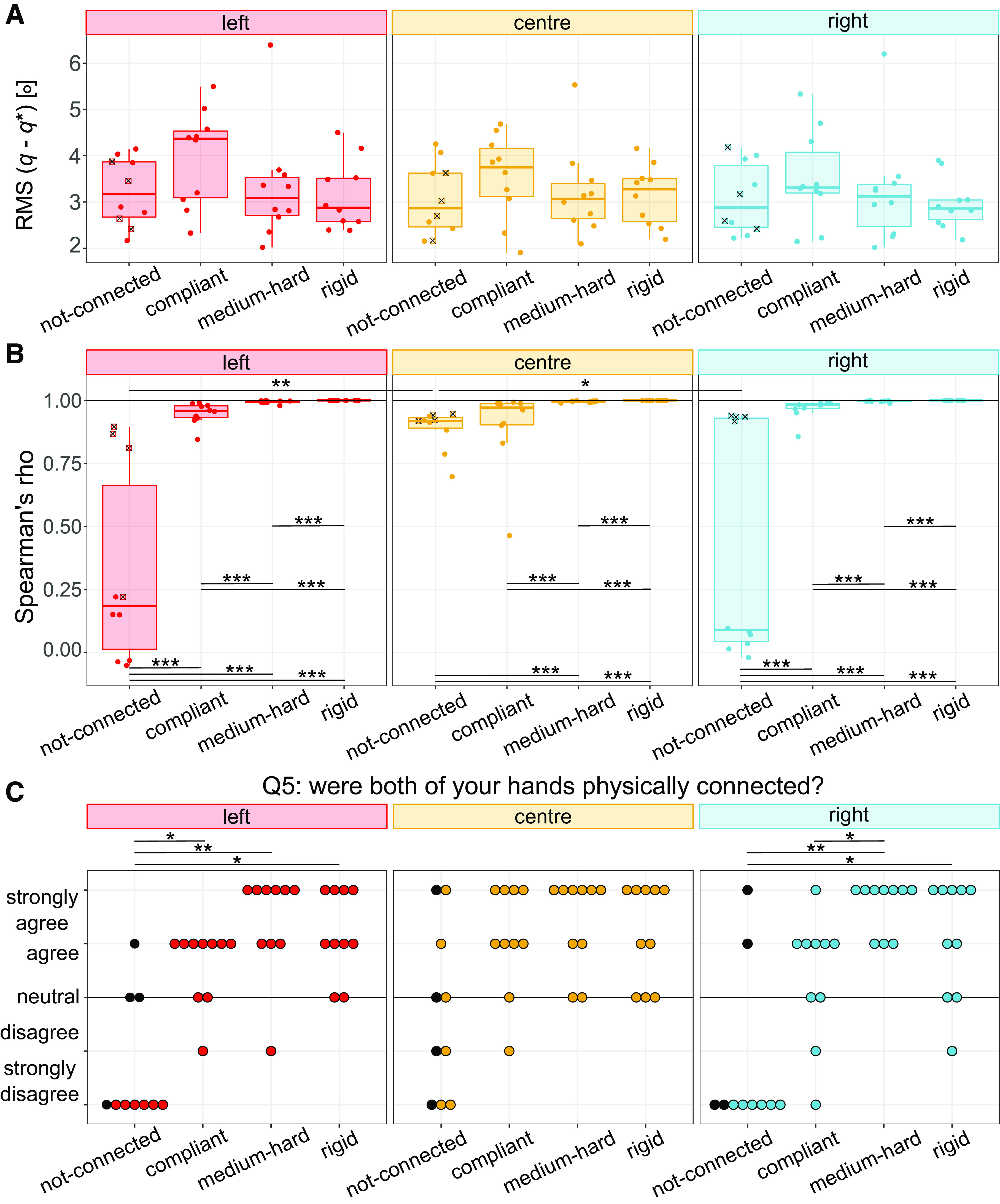
*Experiment 2*: tracking error (*A*) and correlation between the hands’ positions for each experimental condition where each dot is the mean across the last five trials per participant (*B*). *C*: perception of a physical connection. Squared-crossed markers (*A* and *B*) and black dots (*C*) represent participants from the “atypical” subset. It is noted that three of these participants belonged to the “atypical” subset from *experiment 1* (as they belonged to sequence A) and the fourth participant belongs to the data additionally collected for *experiment 2*. In this way, a total of 5/19 participants who tried the not-connected condition across both experiments displayed “atypical” behaviors. *P < 0.05, **P < 0.01, ***P < 0.001. Only significant comparisons are displayed.

Tracking error analysis (Fig. 5*A*) did not reveal a main effect of the connection [*F*(3,36) = 0.95*, P* = 0.43] nor a significant interaction [*F*(6,72) = 1.18*, P* = 0.33]. A main effect was only observed for the cursor weighting [*F*(2,72) = 4.71*, P* = 0.012], where participants were more accurate when the cursor was only influenced by their dominant right hand compared with the left (*W* = 632*, Z* = −2.74*, P* = 0.006).

Participants displayed varied behaviors with a compliant connection, where the correlation between the hands did not significantly differ from participants using a virtual coupling with the center cursor (*U* = 32*, Z* = −1.16*, P* = 0.25) and with the correlation increasing with stiffer connections (Fig. 5*B*, all *P* < 0.001). This suggests that motor behavior may not change with the presence of a mechanical connection, but instead with its strength. However, the not-connected was the only group that showed different behaviors for different cursor conditions (center vs. left: *W* = 0*, Z* = −2.93*, P* = 0.003; center vs. right: *W* = 51*, Z* = −2.29*, P* = 0.02).

##### In the not-connected group, no connection was perceived with the unequal cursor weighting.

Instead, with the center cursor, it was not perceived as being different from any of the mechanical connections (all *P* > 0.6). Although most connected conditions were clearly perceived as having a connection (Fig. 5*C*), this was not the case for the compliant group under the right cursor weighting, which was less clearly perceived as a connection than the medium-hard group (*U* = 14*, Z* = −2.33*, P* = 0.02) and not different from the virtual coupling group (*U* = 20*, Z* = −1.88*, P* = 0.06).

## DISCUSSION

We investigated how healthy right-handed participants coordinate their hands in a redundant bimanual continuous tracking task, and how this coordination is affected by virtual and mechanical coupling. The results of our experiments indicate that both a virtual coupling (via a shared single cursor) and a mechanical connection between the hands can induce participants to move their two hands simultaneously to track a moving target. Participants achieved a performance that did not depend on the coupling type (Fig. 3*B*) or on the stiffness of a mechanical connection (Fig. 5*A*). The effort tended to be balanced among the hands, where only a compliant mechanical connection led to unbalanced contributions, in favor of a more co-contracted right hand (Fig. 4*C*). Interestingly, the effort distribution only changed with the task asymmetry when the hands were not mechanically connected (Fig. 4, *A*–*C*).

### Most Participants Used Their Hands in a Task Relevant Manner (H1)

Despite the participants not being informed of the cursor weighting for the different conditions, both coupling types resulted in them using both hands (Fig. 2*A*). Therefore, most participants only used the hands when they were relevant to the task. They recognized when some movement did not impact the cursor, identified task-relevant feedback, and produced only task-relevant commands. It has been shown that when individuals identify visuomotor discrepancies, which can occur during the integration of their cursor’s visual feedback and their hand’s proprioception (43), the CNS can adapt its response depending on the task relevance (44). In our task, when the cursor weighting changed so that one hand became task irrelevant, some participants showed exploratory motions (see Supplemental Section 1), which may have been a consequence of them identifying and trying to adapt to the visuomotor discrepancies.

These results are consistent with *Hypothesis H1* and align with stochastic optimal control models (24) that predict that the CNS would distribute work between the hands to minimize error and effort, such that a hand would only be used if it contributes to the task (4, 22). Previous work in continuous tasks (i.e., planar tracking) (29) observed participants continuing to produce task-irrelevant motion, possibly because they could not identify the feedback or could not adapt to the given mapping. Our findings contrast with these observations and suggest that the minimization of task-irrelevant motions can still be found in tasks requiring constant hand adjustments.

However, 5/19 participants (see Fig. 5) moved both hands when they were uncoupled. In this case, the “unnecessary” movements of one hand were correlated with those of the hand controlling the cursor (Fig. 3*C*). What could explain this behavior? First, these participants may have missed the sensory cues or failed to reduce task irrelevant commands. For example, *participant ID6* moved the left hand more in the not-connected-right condition while reporting that “more contribution of the right hand” was needed compared with the virtual coupling (Supplemental Figs. S1 and S7). However, an incorrect interpretation of sensory feedback could not explain the behavior of some “atypical” participants, who showed exploratory movements (Supplemental Fig. S2) but reported preferring coordinated motions: “The cursor’s control was easier when I used two hands, I tried using one and it was not as easy” (*ID16*, not-connected-left, Supplemental Fig. S7). Alternatively, these behaviors could reflect the consideration of bimanual coupling related constraints (45, 46). Synchronized symmetric motions [which exploit intrinsic neural coupling via interhemispheric connections (47)] are known to be accurate and stable during bimanual coordination (48, 49).

### The Hands’ Effort Distribution Was Mostly Balanced and Was Only Altered by the Cursor Weighting without a Mechanical Connection (H2)

Contrary to *Hypothesis H2*, the contributions of (virtually or mechanically) coupled hands were balanced (Fig. 2, *B* and *C*), except for a higher right-hand CC in participants with a compliant mechanical connection (Fig. 4, *B* and *C*). Although previous works on virtually coupled isometric tasks (30) would predict a lower contribution of the left/noisier hand (25), our results align with previous findings in virtually coupled planar tracking (29) where the hands’ contributions to a shared cursor’s motion were balanced. Interestingly, during all mechanically connected conditions, the left hand had a higher amount of motion than the right, where its higher intrinsic noise may have caused it to move with less fine control (Fig. 2*A*).

Furthermore, introducing asymmetry by changing the cursor weighting did not affect the effort distribution for any of our mechanically connected conditions, contrary to our expectations. This lack of asymmetry may be caused by participants not being able to identify which hand has the more reliable feedback, which could be due to the hands being too restricted (even for our compliant connection). Alternatively, participants may be less aware of how much motion/effort they are using in each hand.

Overall, we only observed a clear influence of lateralization in the CC imbalance with the compliant connection (Fig. 4*C*). Here, participants may have felt delayed reaction forces and increased their dominant hand’s CC to either rely on the less noisy dominant hand, or to stabilize the cursor movement. This increased CC in the dominant hand has been observed in response to instability for some symmetric (nonredundant) bimanual tasks (37). However, Woytowicz et al. (35, 36) reported a stabilizing advantage of the nondominant hand in nonredundant tasks where asymmetry was introduced by giving specific hand instructions (i.e., one hand to reach and the other to stabilize). This differs from our still redundant asymmetric conditions.

### The Coupling Type Did Not Impact Task Performance (H3)

Against *Hypothesis H3*, the addition of a mechanical connection did not improve tracking accuracy, independently of its stiffness. Therefore, our results differ from findings in nonredundant tasks such as object holding, where haptic feedback improved performance (14). This could be caused by the participants being unaware of the connection, not using the additional feedback or finding that the additional feedback was not beneficial for task performance.

Whenever their hands were mechanically connected, participants felt like their “hands were physically connected” (Fig. 3*D*) and reported “forces” that were perceived as “assistive” (Supplemental Fig. S5). This suggests that they were aware of the connection and considered the feedback to be useful. This was supported by some questionnaire responses (e.g., “I flexed both hands because I think squeezing helped me control better the motion,” *ID12* during connected center, Supplemental Fig. S8).

Therefore, it is likely that the additional haptic feedback did not improve performance as it was not task relevant. This is different from nonredundant bimanual tasks like object holding, where smoothly modulating the distance between the hands directly benefits performance. This also differs to human-human studies in which participants improved their individual performance when mechanically connected to a partner in a common tracking task (18), where the tracking accuracy also increased with the connection stiffness (20). Although in these cases the mechanical connection allowed for the exchange of information in addition to force transfer, the natural interhemispheric connection present in bimanual interaction may already facilitate that exchange.

Despite not affecting performance, additional haptic feedback was preferred (Supplemental Fig. S5) and led to more tightly coupled hand motions, where the stiffer mechanical connections improved the correlation between the hands (Fig. 5*B*). The virtual coupling and the compliant connection led instead to lower correlation values (Fig. 3*C*), which may stem from the variability between the hands’ less constrained motion [as minimizing it would incur additional effort (50)]. In turn, there was a larger variability between participants, who likely used different control strategies. This aligns with findings in both discrete [i.e., reaching (1, 51)] and continuous [i.e., path following (52)] virtually coupled tasks where task-irrelevant variability did not hinder task performance.

In accordance with previous studies, our results show better dominant unimanual tracking (29, 53) (Fig. 3*B*). This was despite the right-hand training being carried out first and given that motor skills learnt by the dominant arm can be transferred to the nondominant (54, 55). This may have impacted *experiment 1*’s performance in the connected-right condition, which was worse than in the not-connected-right. Although this reduced performance may have derived from the added inertia of the mechanically connected nondominant hand, no differences were observed in *experiment 2*, suggesting that the reduced tracking accuracy is not necessarily a result of the mechanical coupling.

### Application Considerations

In summary, both virtual and mechanical coupling induced the two hands to contribute to the task. However, task asymmetry only modulated effort distribution when the hands were not mechanically connected. Interestingly, the performance was similar across all coupling levels, although mechanical coupling was preferred and could induce the hands to move more tightly together.

These findings suggest that a virtual coupling can induce active contributions from both hands without impacting performance. Could this be used to develop simpler training devices to promote the affected hand’s use in individuals with hemiplegia? To answer this, further considerations need to be taken. For example, patients with severe impairments may still require mechanical assistance, such that initially relying on a rigid mechanical connection may be advantageous. However, given rigid modes that constrain the use of redundant solutions may be detrimental to motor learning (8), using more compliant modes could be beneficial in later training stages. Moreover, impaired sensing may prevent the correct identification of the visuomotor mapping, thus resulting in behaviors like those of our atypical subjects. Here, alternative methods to alter effort distribution could be explored, such as vibratory feedback or visual perturbations, which biased muscle use and motor behaviors during virtual coupling (33, 56), or force cues, which reduced nonaffected hand compensation during mechanical coupling (6).

Finally, we would expect stroke survivors to show different lateralized behaviors to controls (57) and to observe lesion-dependent differences in their capabilities to use the task redundancy without impacting their performance (51). Therefore, the aforementioned results need to be tested on the relevant population before deciding on a design for bimanual rehabilitation devices.

## DATA AVAILABILITY

Data will bemade available upon reasonable request.

## SUPPLEMENTAL DATA

10.6084/m9.figshare.21370950 Supplemental Material: https://doi.org/10.6084/m9.figshare.21370950.

## GRANTS

This research was supported by the EPSRC Centre for Intelligent Games and Game Intelligence Grant EP/L015846/1 and by the EC Grants H2020 FETOPEN 899626 NIMA, ICT 871767 REHYB, and ICT 871803 CONBOTS.

## DISCLOSURES

No conflicts of interest, financial or otherwise, are declared by the authors.

## AUTHOR CONTRIBUTIONS

N.P.-P., J.E., E.I., I.F., and E.B. conceived and designed research; N.P.-P. performed experiments; N.P.-P., J.E., and E.I. analyzed data; N.P.-P., J.E., E.I., I.F., and E.B. interpreted results of experiments; N.P.-P. prepared figures; N.P.-P. and J.E. drafted manuscript; N.P.-P., J.E., E.I., I.F., and E.B. edited and revised manuscript; N.P.-P., J.E., E.I., I.F., and E.B. approved final version of manuscript.
